# *Dendrogramma*, New Genus, with Two New Non-Bilaterian Species from the Marine Bathyal of Southeastern Australia (Animalia, Metazoa *incertae sedis*) – with Similarities to Some Medusoids from the Precambrian Ediacara

**DOI:** 10.1371/journal.pone.0102976

**Published:** 2014-09-03

**Authors:** Jean Just, Reinhardt Møbjerg Kristensen, Jørgen Olesen

**Affiliations:** Section of Biosystematics, Natural History Museum of Denmark (Zoological Museum), University of Copenhagen, Copenhagen, Denmark; Sars International Centre for Marine Molecular Biology, Norway

## Abstract

A new genus, *Dendrogramma,* with two new species of multicellular, non-bilaterian, mesogleal animals with some bilateral aspects, *D. enigmatica* and *D. discoides*, are described from the south-east Australian bathyal (400 and 1000 metres depth). A new family, Dendrogrammatidae, is established for *Dendrogramma*. These mushroom-shaped organisms cannot be referred to either of the two phyla Ctenophora or Cnidaria at present, because they lack any specialised characters of these taxa. Resolving the phylogenetic position of *Dendrogramma* depends much on how the basal metazoan lineages (Ctenophora, Porifera, Placozoa, Cnidaria, and Bilateria) are related to each other, a question still under debate. At least *Dendrogramma* must have branched off before Bilateria and is possibly related to Ctenophora and/or Cnidaria. *Dendrogramma*, therefore, is referred to Metazoa *incertae sedis*. The specimens were fixed in neutral formaldehyde and stored in 80% ethanol and are not suitable for molecular analysis. We recommend, therefore, that attempts be made to secure new material for further study. Finally similarities between *Dendrogramma* and a group of Ediacaran (Vendian) medusoids are discussed.

## Introduction

**Figure 1:**
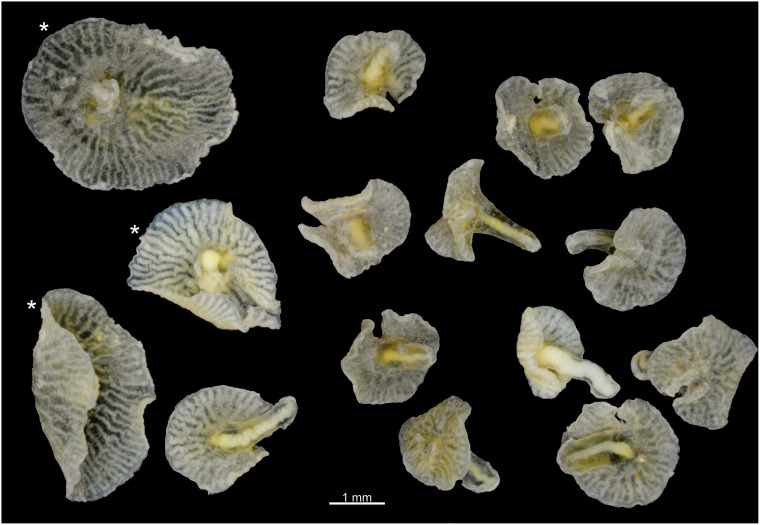
*Dendrogramma* gen. nov., all 15 paratypes of *D. enigmatica* and (with *) *D. discoides.* Photographs taken after shrinkage (see Material and Methods).

**Figure 2:**
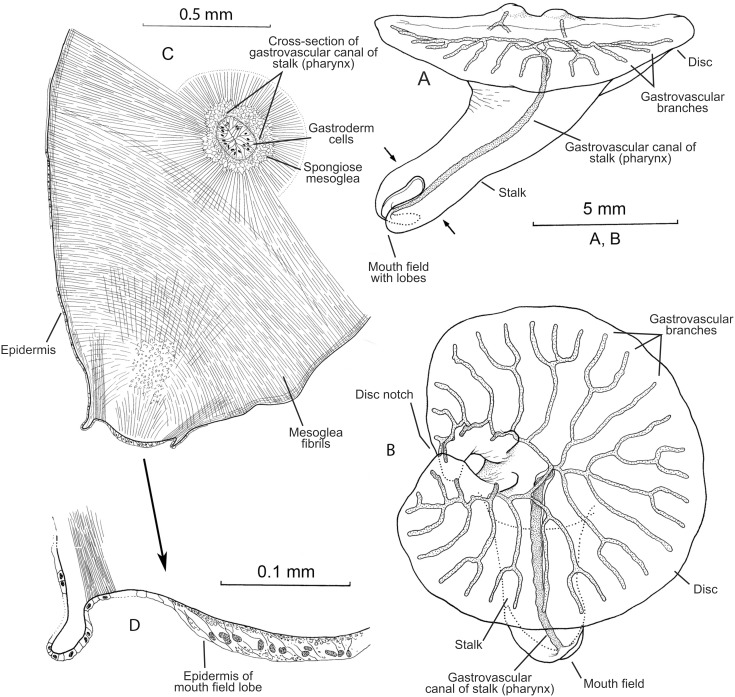
*Dendrogramma enigmatica* sp. n., A, holotype, ‘lateral’ view. B, same, aboral view. C, cross-section through approx. half of stalk (level indicated by arrow heads in Fig. 2A) showing gastrovascular canal in centre (pharynx), mouth-field lobe with thickened epidermis, and main systems of fibrils in mesoglea. D, enlargement of mouth-field lobe. Drawings made before shrinkage.

**Figure 3:**
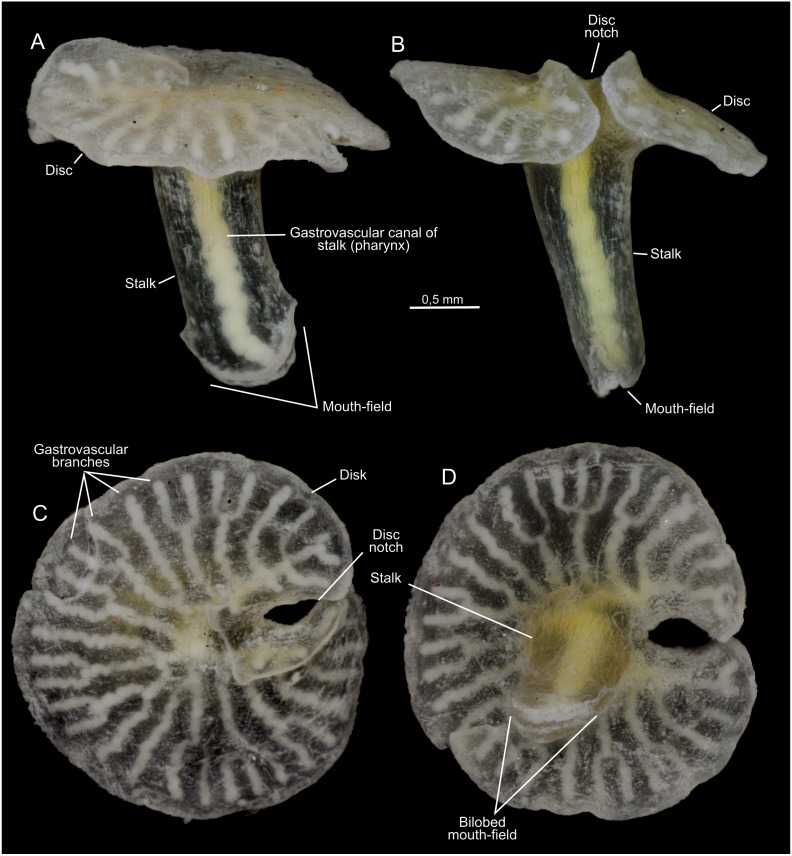
*Dendrogramma enigmatica* sp. nov., holotype. A, B, lateral views; C, aboral view, D, adoral view. Photographs taken after shrinkage.

**Figure 4:**
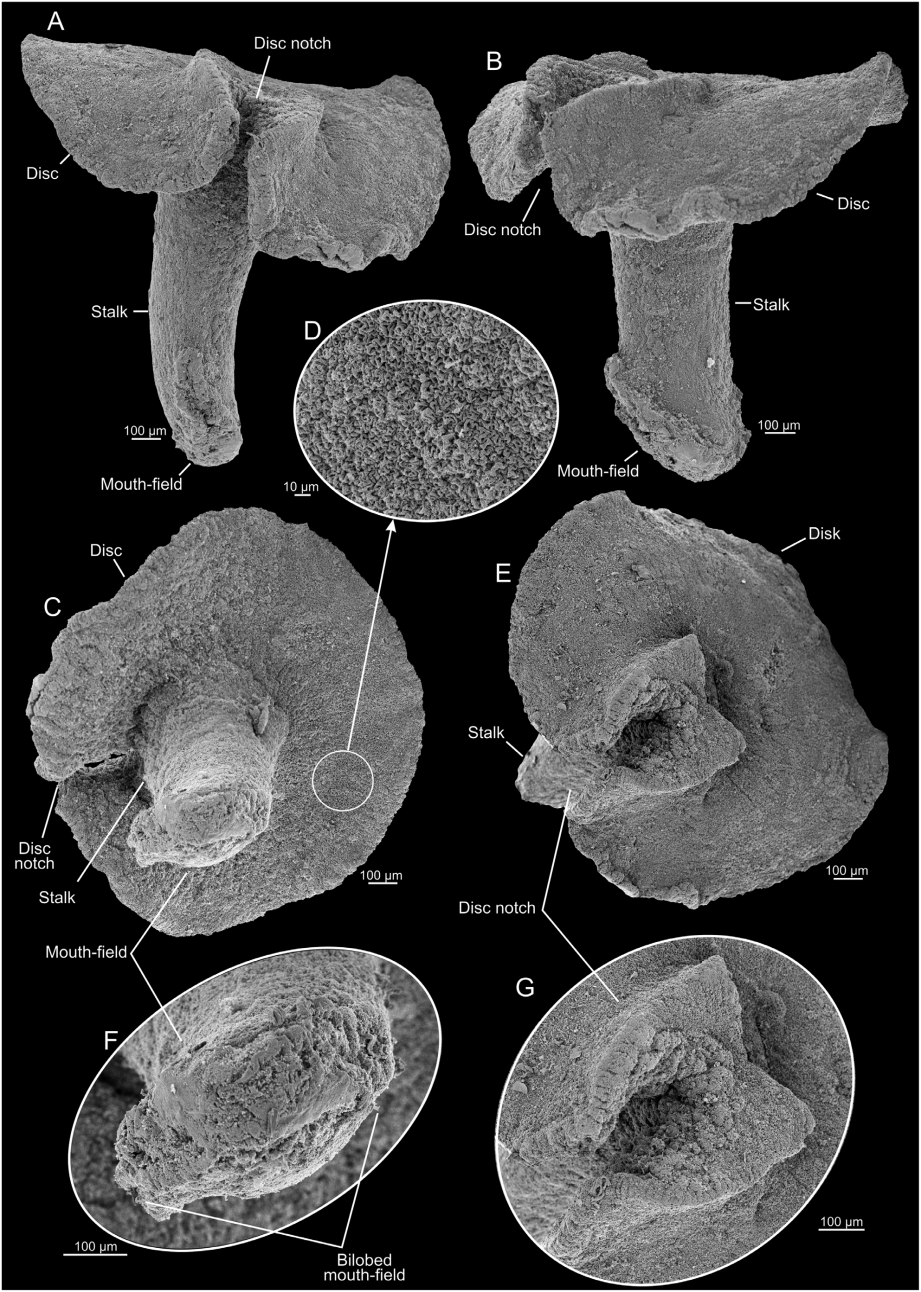
*Dendrogramma enigmatica* sp. nov., paratype. A, B, lateral views; C, adoral view; D, close-up of disc surface; E, aboral view; F, tip of stalk; G, aboral view of notch of disc surface. SEM micrographs made after shrinkage.

**Figure 5:**
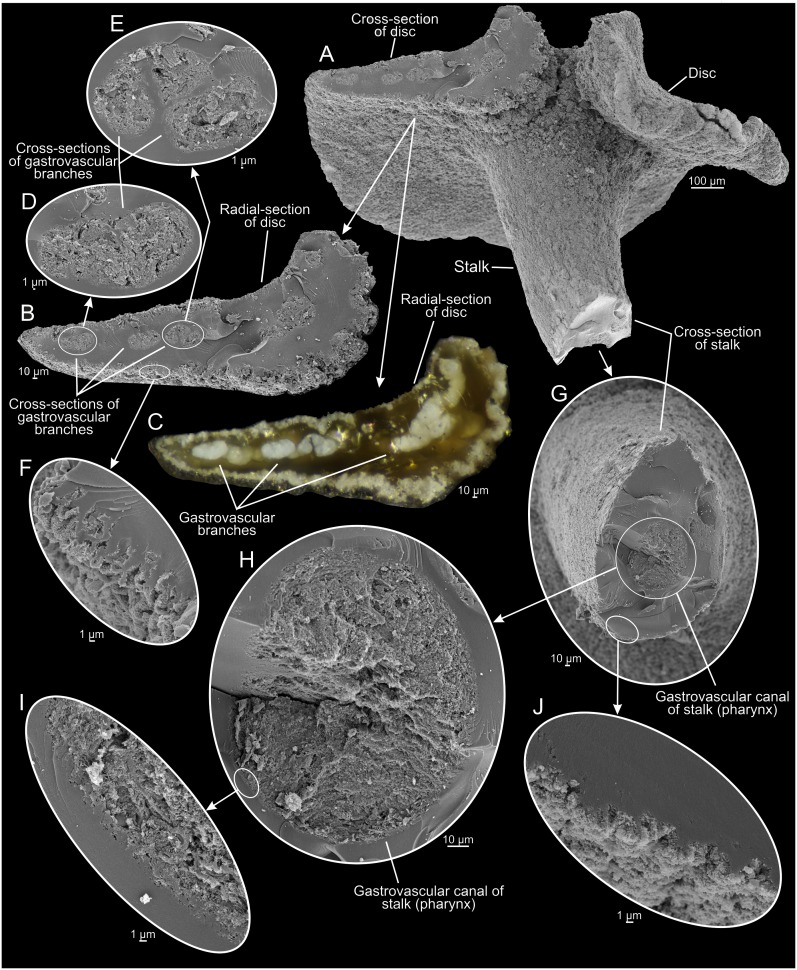
*Dendrogramma enigmatica* sp. nov., paratype, same specimen as in **figure 4** after cutting away part of stalk and disc; A, lateral view. B, radial-section of disc; C, radial-section of disc in light microscopy before sputter coating for SEM. D,E, cross-sections of gastrovascular branches in disc. F, epidermis of disc. G, cross-section of stalk. H, cross-section of gastrovascular canal of stalk (pharynx). I, gastrodermis of gastrovascular canal (pharynx) of stalk. J, epidermis of stalk. SEM micrographs made after shrinkage.

**Figure 6:**
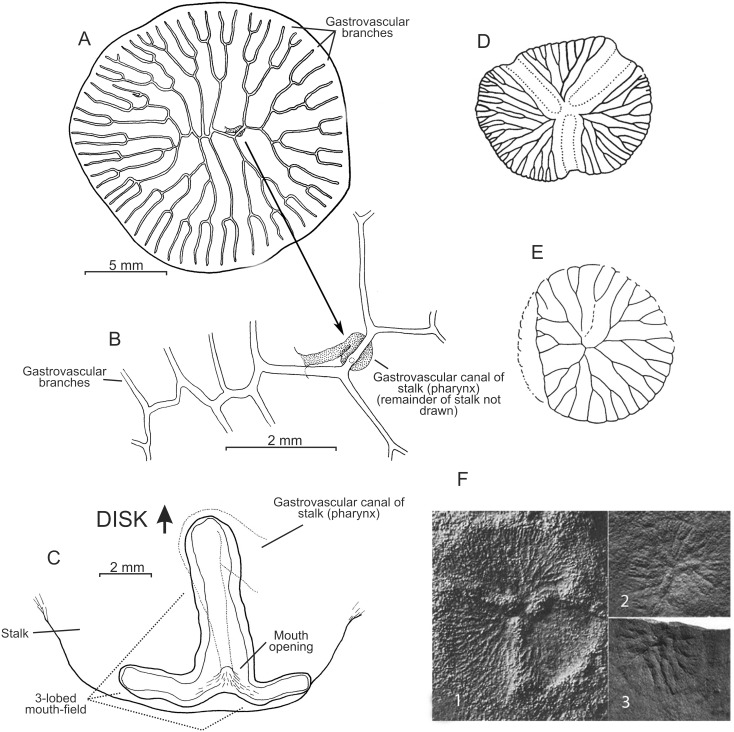
*Dendrogramma discoides* gen. et sp. n., A, holotype, aboral view. B, enlargement of A showing gastrovascular canal (stippled) of stalk (pharynx) and point of connection to the first branching node of gastrovascular system of the disc. C, paratype, oblique oral view of trilobed mouth-field with mouth opening in centre; entire pharyngeal part of the gastrovascular system is shown. D, *Albumares* with trilobed field (reproduced from [19]). E, *Rugoconites tenuirugosus* (reproduced from [19]). F, 1. *Albumares brunsae*, 2. *Anfesta stankovskii,* 3. *Rugoconites enigmaticus*; (all three from [23]; sizes: see text in Discussion). Drawings of *Dendrogramma* made before shrinkage.

**Figure 7:**
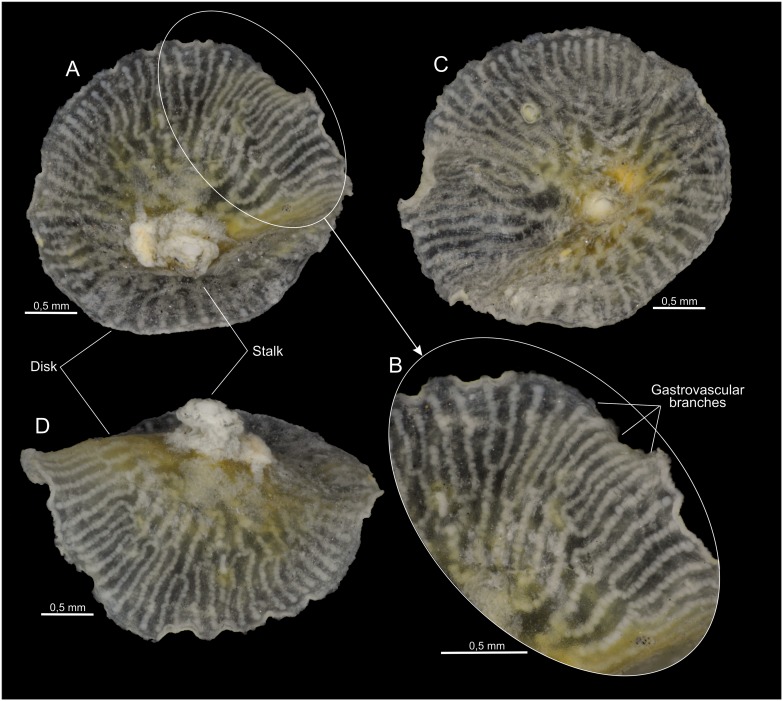
*Dendrogramma discoides* gen. et sp. n., various aspects of holotype. A, adoral view. B, enlarged part of disc; C, aboral view; D, oblique adoral view. Photographs taken after shrinkage.

The aim of this paper is to present a group of non-bilaterian metazoan organisms that cannot at present be placed in an existing phylum. The two species described, *D. enigmatica* and *D. discoides* (Figs 1–7) in new genus *Dendrogramma* of the new family Dendrogrammatidae were collected at 400 and 1000 metres on the Australian continental slope off eastern Bass Strait and Tasmania during a cruise in 1986. The first author subsequently worked up the entire material at Museum Victoria, Melbourne, Australia. The specimens in question were not recognised in the field, but were extracted from bulk samples in the laboratory during sorting (see further under Material and Methods below).

For reasons given in the Discussion we cannot refer these specimens to Ctenophora or Cnidaria, two phyla that are often considered diploblastic [1] and bear some resemblance to *Dendrogramma*. We therefore place the new genus as *incertae sedis* in the Metazoa, pending the collection of more material. We do, however, discuss some possible phylogenetic implications and draw attention to similarities between *Dendrogramma* and some fossil medusoids from the Precambrian Ediacara (Vendian) fauna [2].

## Materials and Methods

### Sampling and preparation

The material was collected with a WHOI epibenthic sled with closing devise operated at the bottom for a distance of approximately 250–300 metres. Sampling was undertaken from the Australian National Facility Research Vessel ORV Franklin. No special permission was required to collect benthic bathyal invertebrates in the area. Samples were washed through a stack of successively finer sieves following removal of large organisms (e.g., echinoderms, decapods, fish), and large shells and stones. The resulting material was bulk fixed in neutral (Borax) formalin. Later, in the laboratory, the bulk samples were washed in water and transferred to 80% ethanol. The animals described are not listed as endangered.

After sorting, when the unusual nature and uncertain taxonomic affinity of these organisms became apparent, the remainder of the bulk samples from the relevant 1986 stations (32 and 48) was revisited to search for material that might be related to the unknown organisms. None was found which is in accordance with the fact that not a single one of the 18 specimens showed signs of having been torn off either a hard substrate or a biological (common/colonial) base.

No additional specimens were found in a subsequent cruise to the same general area in 1988 in which the first author participated.

The sediment of the deeper sample (1000 m) consisted of relatively fine calcareous rubble mixed with mud and clay; that of the shallower one (400 m) consisted of coarse calcareous fragments (e.g., mollusc shell, bryozoans) mixed with mud.

The two holotypes (Figs 2, 6A–B) were drawn under dissecting microscope with a camera lucida by the first author shortly after discovery. Details in Fig. 2 of hematoxylin and eosin (HE) stained paraffin sections were drawn under a compound microscope (Olympus BH-2) also with camera lucida using phase contrast and Nomarski (Fig. 2C,D). These sections have since bleached preventing further study. Subsequently the samples were brought to Canberra with the first author, where it was later found that they were close to drying out. Unfortunately absolute alcohol was provided without comment instead of the requested 80% ethanol, resulting in immediate strong shrinkage of the specimens which were, furthermore, rendered glassy brittle. These specimens are, however, clearly recognisable as one or the other of the two new species (see Fig. 1). The photographs in Figures 1, 3, 5C, 7 were taken with a Nikon D700 fitted to an Olympus SZX10 dissecting microscope and operated via the software ControlMyNikon v. 4.3. To cover a larger depth of field, each published image is the result of several photographs taken at different focal points which were combined with Zerene Stacker v. 1.04. One entire paratype specimen of *Dendrogramma enigmatica* was prepared for SEM (dehydrated, critical point dried, metal coated) and observed in a JEOL JSM-6335F (Fig. 4). Cross sections of the already SEM mounted specimen was made of both the cylindrical stalk and the disc to investigate internal structures (Fig. 5). Before re-coating for SEM one light microscopy image was made of the cut surface of the disc which shows the internal distribution the gastrovascular branches in the disc and the mesoglea (Fig. 5C).

The family diagnosis and species descriptions are short and based on the original illustrations. The majority of the material is lodged at Museum Victoria (NMV numbers), Melbourne, Australia but paratypes are deposited at the Natural History Museum (ZMUC numbers) of Denmark, Copenhagen, Denmark.

### Nomenclatural Acts

The electronic edition of this article conforms to the requirements of the amended International Code of Zoological Nomenclature, and hence the new names contained herein are available under that Code from the electronic edition of this article. This published work and the nomenclatural acts it contains have been registered in ZooBank, the online registration system for the ICZN. The ZooBank LSIDs (Life Science Identifiers) can be resolved and the associated information viewed through any standard web browser by appending the LSID to the prefix “http://zoobank.org/”. The LSID for this publication is: urn:lsid:zoobank.org:pub: DFFC9FC7-61B2-412E-BDA0-641F1AD998D3. The electronic edition of this work was published in a journal with an ISSN, and has been archived and is available from the following digital repositories: PubMed Central and LOCKSS.

## Results

### Metazoa

It has been suggested during review that *Dendrogramma* could represent a new non-bilaterian phylum. While we may agree, we refrain from erecting such a high-level taxon for the time being, because new material is needed to resolve many pertinent outstanding questions.

### Dendrogrammatidae, new family

urn:lsid:zoobank.org:act:73DFB28C-EF41-48F7-B324-09503D79B382.

#### Diagnosis

Multicellular, mesogleal, apparently diploblastic animal. Body divided into cylindrical stalk and broad, flat disc (Figs 2A, B, 3, 4, 5A, 7). Simple round mouth opening situated in slightly depressed lobed field on rounded apex of stalk. With gastrovascular system comprising a simple tube centrally in stalk (pharynx) running from mouth to base of disc, then branching dichotomously, including first branching node (Fig. 6B), in disc at right angles to stalk. Epidermis composed of single layer of low, uniform cells; gastrodermis composed of single layer of elongate, vacuolated cells tapering towards narrow gastrovascular canal (pharynx) (Fig. 2C); epidermis of mouth-field lobes with thickened, elongate, apparently vacuolated/glandular cells (Fig. 2D). Dense mesoglea milky translucent when formalin fixed except for refractive sheath of spongiose mesoglea surrounding gastrodermis of gastrovascular canal in stalk (pharynx) (Figs 2C, 5C). Mesoglea criss-crossed by fibrils including cylindrical sheet under epidermis (Fig. 2C, D).

#### Component genus

*Dendrogramma*, new genus.

### Dendrogramma, new genus

urn:lsid:zoobank.org:act:4D13A8A6-8768-4103-AA81-9772D0D0F39E.

#### Diagnosis

With the characters of the family.

#### Etymology

The name of the genus alludes to the branching pattern of the gastrovascular system of the disc.

#### Type-species

*Dendrogramma enigmatica* new species.

#### Additional species

*Dendrogramma discoides* new species.

### Dendrogramma enigmatica new species

urn:lsid:zoobank.org:act:9BBD1C77-4B5B-4248-8B0D-79D598F07E05.

Figs 1–5.

#### Holotype

Australia, Victoria, S of Pt. Hicks, 38° 21.9′S 149° 20.0′E–38° 21.40′S 149°20.90′E, 1000 m, WHOI epibenthic sled, RV *Franklin* Stn SLOPE 32, 23 July 1986, G.C.B. Poore et al., NMV F65709.

#### Paratypes

9 specimens, same data as holotype, NMV F60459. 2 specimens, same data as holotype, ZMUC-DEN-01. 1 specimen used for SEM, same data as holotype, ZMUC-DEN-02. 1 specimen [fragments], Australia, Tasmania, off Freycinet Peninsula, 41° 57.50′S 148° 37.90′E, 400 m, coarse shell, WHOI epibenthic sled, RV *Franklin* Stn SLOPE 48, 27 July 1986, M.F. Gomon et al., NMV F60458.

#### Description (holotype)

Tapering stalk elongate, about 7/10 as long as disc diameter; length approximately 1.5 width at base of disc. Disc diameter approximately 11 mm (2.8 mm after shrinkage), stalk length approximately 7.8 mm (cf. Fig. 2) (2 mm after shrinkage). Disc nearly circular with single marginal notch and small rounded hump on each side of notch on disc surface. Mouth-field asymmetrically bilobed, reaching farther up one side of the stalk than the other. Disc with 37 terminals of the gastrovascular branches.

#### Remarks

*Dendrogramma enigmatica* sp. nov. differs from the following species by its much longer stalk with a bilobed mouth field, and a marginal notch in the disc.

#### Etymology

This species has been and still is a great enigma.

### Dendrogramma discoides new species

urn:lsid:zoobank.org:act:4EDB8115-4138-4682-9A79-76D8DFB19650.

Figs. 1, 6–7.

#### Holotype

Australia, Victoria, S of Pt. Hicks, 38° 21.9′S 149° 20.0′E–38° 21.40′S 149°20.90′E, 1000 m, WHOI epibenthic sled, RV *Franklin* Stn SLOPE 32, 23 July 1986, G.C.B. Poore et al., NMV F65710.

#### Paratypes

2 specimens, data as holotype, NMV F65711. 1 specimen, data as holotype, ZMUC-DEN-03.

#### Description (holotype)

Stem short, length approximately 1/10 disc diameter; not longer than wide at base of disc. Disc diameter approximately 17 mm (3 mm after shrinkage), stalk length approximately 4.5 mm (cf. Fig. 6 C). Disc circular with entire margin. Mouth-field of three lobes, two lobes of equal length both longer than the third lobe. Disc with 63 terminals of the gastrovascular branches.

#### Remarks

*Dendrogramma discoides* sp. nov. differs from *D. enigmatica* by its much shorter stalk and entire disc.

#### Etymology

This species is named for the shape of the disc.

## Discussion

The two *Dendrogramma* species are multicellular (metazoans), non-bilaterian, apparently diploblastic animals with a dense mesoglea between an outer epidermis and an inner gastrodermis. The animals are composed of a body divided into a stalk with a mouth opening terminally, and a flattened disc. The mouth is set in a specialised, lobed epidermis field, leading into a gastrodermis-lined gastrovascular canal (pharynx) in the stalk which aborally branches dichotomously into numerous radiating canals in the disc. While the animals are certainly multicellular, the precise structural identity of the epithelia lining the gastrovascular canal and the external remain to be studied and compared to that of other metazoans.

*Dendrogramma* shares a number of similarities in general body organisation with the two phyla, Ctenophora and Cnidaria, but cannot be placed inside any of these as they are recognised currently. We can state with considerable certainty that the organisms do not possess cnidocytes, tentacles, marginal pore openings for the radiating canals, ring canal, sense organs in the form of e.g., statocysts or the rhopalia of Scyphozoa and Cubozoa, or colloblasts, ctenes, or an apical organ as seen in Ctenophora. No cilia have been located. We have not found evidence that the specimens may represent torn-off parts of colonial Siphonophora (e.g., gastrozooids). Neither have we observed any traces of gonads, which may indicate immaturity or seasonal changes. No biological information on *Dendrogramma* is available. To judge from their construction, both species appear unable to swim (the ‘disc’ appears inflexible in preserved specimens). With their small, simple mouth opening it would seem likely that they feed on micro-organisms, perhaps trapped by mucus from the specialised lobes surrounding the mouth opening.

Although *Dendrogramma* cannot at present be referred to Ctenophora or Cnidaria, those two phyla share more characteristics with *Dendrogramma* than does any other phylum. These include the presence of apparently only two germ layers (diploblastic) with a mesoglea in between (but see [3] for discussion of a third germ layer in Ctenophora), and the presence of a single mouth opening through which food is ingested and waste is released, leading into a gastrovasular cavity becoming highly branched terminally. It is therefore possible that *Dendrogramma* may eventually find a phylogenetic position as closely related to one of these phyla (e.g., as sister group), but at present no unique characters suggesting close affinity to any of these has been found (see above).

**Figure 8:**
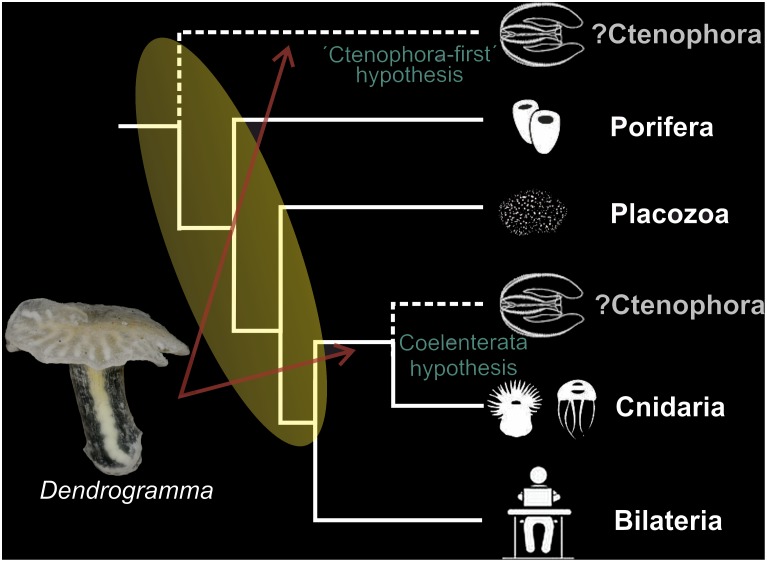
Possible positions of *Dendrogramma* in a simplified phylogeny showing the deepest splits in the metazoan Tree of Life. The position of Ctenophora is controversial so two possibilities have been shown with dashed lines, one as sister group to the remaining metazoans (the ‘Ctenophora-first’ hypothesis), and one as sister group to Cnidaria (Coelenterata hypothesis) (based on [6], [7], [8], [24], [25]. We suggest that *Dendrogramma* most likely is related to Ctenophora and/Cnidaria (red arrows) due to general similarities in body organisation (see Discussion). However, depending on the position of Ctenophora and on whether certain aspects of *Dendrogramma* (e.g., mesoglea and gastrovascular system) are ancestral for Metazoa or modified, *Dendrogramma* can be positioned in a variety of ways below Bilateria (yellow oval).

The question of the phylogenetic position of *Dendrogramma* also depends on how the basal metazoan lineages are related to each other, a question which can be reduced to considering the relationship between Porifera, Placozoa, Cnidaria, Ctenophora, and Bilateria [4], [5]. In theory 105 possibilities exist for grouping five taxa, but only few have in this case actually been suggested [5]. The traditional view is that Porifera is sister group to all other animals, a view based on their lack of tissue organisation, lack of nervous system, and the similarities of choanocytes to choanoflagellates [3] (Fig. 8). However, a recent finding, supported by much molecular data (incl. genomic data for all major taxa), proposes that Ctenophora, rather than Porifera, is sister group to all other metazoans [6], [7], [8], [9]. Due to this significant conflict regarding deep metazoan phylogeny, we have chosen to illustrate possible positions of *Dendrogramma* on a phylogeny with two different positions of Ctenophora (Fig. 8). Regardless of the position of Ctenophora, we suggest that the most likely position of *Dendrogramma* is before Bilateria, being related to either Ctenophora and/or Cnidaria based on the general similarities in body organisation (e.g., presence of mesoglea and gastrovascular system). If indeed Porifera is the sister group to the remaining metazoans as traditionally perceived and recently supported by a re-analysis [10], [11] of a major molecular dataset from [6], [7], and if Ctenophora and Cnidaria are sister taxa ( = Coelenterata), as was the result of the same re-analysis [10], [11], then *Dendrogramma* may be related to Coelenterata.

Ctenophora positioned as sister group to the remaining Metazoa (‘Ctenophora-first’ hypothesis) has recently been supported by adding the genome of a second ctenophore as well as the transcriptome of several other ctenophores [9]. In the same study it was suggested that neural systems in ctenophores evolved independently from those in other animals. If indeed Ctenophora and Cnidaria are placed widely separated (see Fig. 8), it is likely that also the general body organisation of Ctenophora and Cnidaria has evolved in parallel from a poriferan or placozoan-like ancestor. Then the most likely position of *Dendrogramma* would be as sister group to either Ctenophora or Cnidaria based on the similarities in general body organisation. In this way the lack of tissue organisation and nervous system (etc.) in Porifera would be original attributes. However, if the similarities between Ctenophora and Cnidaria regarding general body organisation are homologous (but symplesiomorphic), then this significantly broadens the spectrum of possible phylogenetic positions of the mesogleal *Dendrogramma*. Then, all that can be said is that *Dendrogramma* should be placed somewhere before the Bilateria (yellow area in phylogeny in Fig. 8). In this scenario a multitude of possible positions exist, including sister group to any of the involved taxa (incl. Ctenophora or Cnidaria), and even a position as sister group to the remaining Metazoa is possible.

In summary, the available information about *Dendrogramma* does not allow for a more precise phylogenetic position than being before the Bilateria, possibly on either the lineage leading to the Ctenophora and/or Cnidaria. A more robust phylogeny between the five basal metazoan lineages (Ctenophora, Placozoa, Porifera, Cnidaria, Bilateria) is needed before strong conclusions on the evolution of important characters (such as number of germ layers and presence of nervous system) can be made. In addition, fresh material of *Dendrogramma* appropriately fixed for molecular (genomic) studies, ultra structure, and histology together with additional biological information, if possible, should be obtained before a proper phylogenetic placement can be made. From a morphological point of view, detailed information about the epithelial structure, composition of the mesoglea, nervous system, and muscles fibres (if present) are particularly wanted for comparison with other non-bilaterians.

It is widely thought that bilateral symmetry evolved in the common ancestor of Bilateria, but it has long been known that some members of Cnidaria also exhibit bilateral symmetry [12]. Based on studies of *Hox* genes it has been suggested that bilateral symmetry already evolved before the Cnidaria diverged from Bilateria [12], [13]. The considerable difference in global symmetry between the two species of *Dendrogramma* is additional evidence that symmetry is highly plastic. Both species of *Dendrogramma* exhibit bilateral aspects notably in the lobed field surrounding the mouth opening and in the initial dichotome branching node of the radiating canals. The disc of *D. enigmatica* is clearly bilateral as indicated by the disc notch. The distal extremity of the stalk of *D. discoides* can be interpreted as triradial in which case the unequal length of the lobes of the mouth field is just localised bilateral symmetry. The disc of *D. discoides* may be interpreted as radial symmetric. Considering the differences in symmetry pattern between the two species, even in different parts of the body, the issue of the origin of bilateralism may add additional interest to the study of new material of *Dendrogramma*.

Finally, we would like to point to an interesting similarity between *Dendrogramma* and a small group of Precambrian Ediacara (Vendian) trilobozoid medusoids. In particular we draw attention to taxa such as *Albumares*, *Anfesta*, and *Rugoconites* (the last mentioned is not included in the Trilobozoida by all authors). All three have dichotomously branching radiating canals in a disc. *Rugoconites tenuirugosus* Wade, 1972 (Figs 6 E and F3; size range in the two richest *Rugoconites* beds: 9–29 mm, see [14) appears to be seen in aboral view with a presumed triradiate initial central branching node. This may well be an artefact. If *Dendrogramma* were to be fossilised in the same position it would most probably exhibit the same pattern, in spite of the central (first) branching node being actually dichotomous. *Albumares brunsae* Fedonkin, 1976 (Fig. 6 D, F1; size range 8–15 mm) and *Anfesta stankovskii* Fedonkin, 1984 (Fig. 6 E2; size range 5–18 mm) both possess a trilobed field radiating from the centre, similar to the adoral lobed field of *Dendrogramma discoides*. In view of the considerable depth at which the *Dendrogramma* species were collected we note that the Ediacaran fauna (including some medusoids) of several Canadian locations, e.g. Newfoundland and the Mackenzie Mountains, appear to have lived at bathyal depth to more than 1000 meters [2]. We are aware that the similarities to some of the Ediacaran forms may be independent responses to the same environmental necessities, rather than being evidence of homology. But, if indeed the similarities between *Dendrogramma* and Ediacaran forms such as *Albumares*, *Anfesta*, and *Rugoconites* (Fig. 6E–F) are indicators of close relationship, it has interesting phylogenetic implications and may throw light on the origin of these Ediacaran taxa. Then, if *Dendrogramma* is an off split of either the lineage leading to Ctenophora and/or to Cnidaria (Fig. 8), as suggested by us, *Albumares*, *Anfesta*, and *Rugoconites* would also be in such a position and should therefore be considered ingroup metazoans rather than being a member of a monophyletic extinct kingdom ‘Vendozoa’. The latter taxon has been suggested to be a failed experiment with multicellularity independent of that of the ‘true’ Metazoa [15]. A metazoan affinity of many Ediacaran forms was suggested already early (e.g., as cnidarians or echinoderms, see [16]), a notion that have been supported lately for a number of taxa such as *Tribrachidium* (as a sponge or ctenophore-type organism), *Kimberella* (mollusc), or *Dickinsonia* (early placozoan) [17,18,19,20,21 22]. The possibility of the Ediacaran taxa *Albumares*, *Anfesta*, and *Rugoconites* being true metazoans as mentioned above, based indirectly on the presumed position of *Dendrogramma* is therefore in line with this more recent phylogenetic treatment of various Precambrian Ediacaran forms.
